# 1-(4-Methyl­phen­yl)-2-[4-(trifluoro­methyl)phen­yl]-1*H*-phenanthro[9,10-*d*]imida­zole

**DOI:** 10.1107/S1600536813009471

**Published:** 2013-04-13

**Authors:** T. Mohandas, R. Sathishkumar, J. Jayabharathi, A. Pasupathy, P. Sakthivel

**Affiliations:** a Department of Physics, Shri Angalamman College of Engineering and Technology, Siruganoor, Tiruchirappalli 621 105, India; b Annamalai University, Annamalainagar, Chidambaram, India; c Department of Chemistry, Urumu Dhanalakshmi College, Tiruchirappalli, Tamilnadu, India; d Department of Physics, Urumu Dhanalakshmi College, Tiruchirappalli, Tamilnadu, India

## Abstract

In the title compound, C_29_H_19_F_3_N_2_, the tetra­cyclic ring system is essentially planar [maximum deviation from the best plane = 0.076 (1) Å] and makes dihedral angles of 78.10 (5) and 33.71 (4)° with the methyl­phenyl and fluoro­phenyl rings, respectively. An intra­molecular C—H⋯π inter­action occurs. In the crystal, pairs of C—H⋯π inter­actions link inversion-related mol­ecules.

## Related literature

For background to organic electroluminescent materials and devices, see: Adachi *et al.* (1995 ▶); Loy *et al.* (2002 ▶) and for the photophysical, electrochemical and mobility properties of phenanthro­imidazole derivatives, see: Yuan *et al.* (2011 ▶). For applications of imidazole and phenanthrolene derivatives, see: Moylan *et al.* (1993 ▶); Bu *et al.* (1996 ▶); Wang *et al.* (2002 ▶).
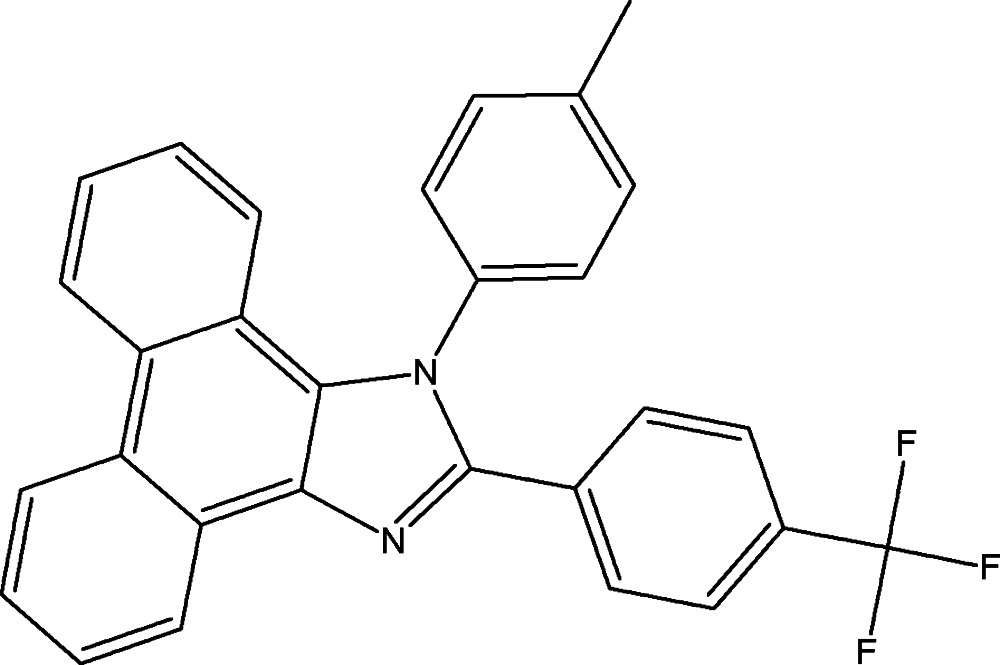


## Experimental

### 

#### Crystal data

C_29_H_19_F_3_N_2_
*M* *_r_* = 452.46
Triclinic, 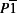

*a* = 8.113 (3) Å
*b* = 11.733 (5) Å
*c* = 12.713 (2) Å
α = 76.397 (1)°
β = 73.490 (2)°
γ = 86.185 (5)°
*V* = 1127.7 (7) Å^3^
*Z* = 2
Mo *K*α radiation
μ = 0.10 mm^−1^
*T* = 293 K
0.35 × 0.30 × 0.25 mm

#### Data collection

Bruker Kappa APEXII CCD diffractometer
Absorption correction: multi-scan (*SADABS*; Bruker, 2008 ▶) *T*_min_ = 0.967, *T*_max_ = 0.977
22540 measured reflections
22540 independent reflections
14735 reflections with *I* > 2σ(*I*)

#### Refinement

*R*[*F* ^2^ > 2σ(*F* ^2^)] = 0.068
*wR*(*F* ^2^) = 0.236
*S* = 1.04
22540 reflections
310 parameters
H-atom parameters constrained
Δρ_max_ = 0.72 e Å^−3^
Δρ_min_ = −0.38 e Å^−3^

### 

Data collection: *APEX2* (Bruker, 2008 ▶); cell refinement: *APEX2* and *SAINT* (Bruker, 2008 ▶); data reduction: *SAINT*; program(s) used to solve structure: *SHELXS97* (Sheldrick, 2008 ▶); program(s) used to refine structure: *SHELXL97* (Sheldrick, 2008 ▶); molecular graphics: *PLATON* (Spek, 2009 ▶); software used to prepare material for publication: *PLATON*.

## Supplementary Material

Crystal structure: contains datablock(s) global, I. DOI: 10.1107/S1600536813009471/go2084sup1.cif

Structure factors: contains datablock(s) I. DOI: 10.1107/S1600536813009471/go2084Isup2.hkl

Additional supplementary materials:  crystallographic information; 3D view; checkCIF report

## Figures and Tables

**Table 1:** Hydrogen-bond geometry (Å, °) *Cg*1 and *Cg*2 are the centroids of the C15–C20 and C8–C13 rings, respectively.

*D*—H⋯*A*	*D*—H	H⋯*A*	*D*⋯*A*	*D*—H⋯*A*
C12—H12⋯*Cg*1	0.93	2.84	3.698 (2)	155
C26—H26⋯*Cg*2^i^	0.93	2.86	3.487 (2)	125

Symmetry code: (i) 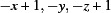.

**Fig. 1.**
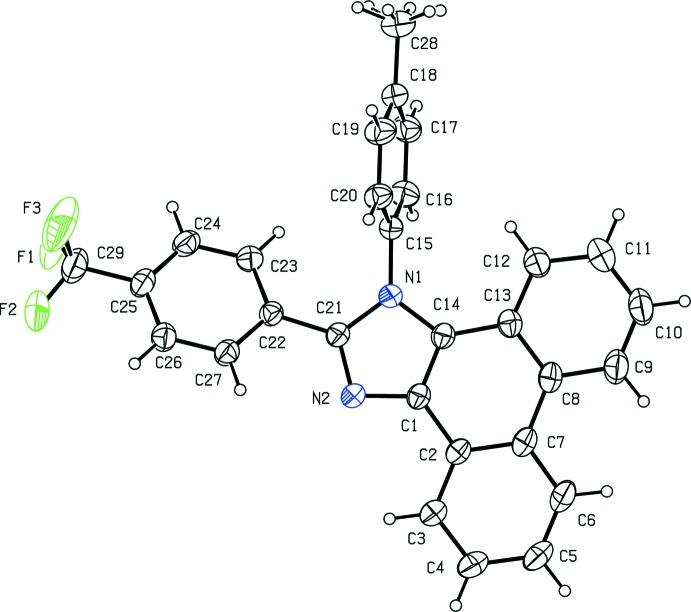
The molecular structure and labelling scheme for (I) with displacement ellipsoids for non-H atoms are drawn at the 30% probability level.

**Fig. 2.**
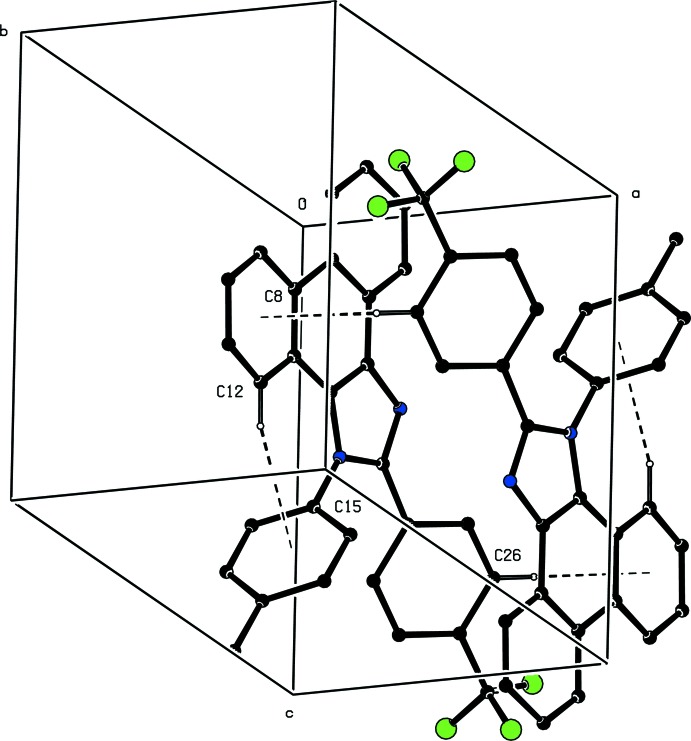
A packing diagram for (I) showing the intramolecular interaction and the centrosymmetrically linked pair of molecules. Dashed lines indicate C—H···π interactions. Hydrogen atoms not involved in the interactions are omitted for clarity.

## supplementary crystallographic information

### Comment

The optical and conductive properties of the conjugated materials containing imidazole and phenanthroline heterocycles have found in many applications (Moylan *et al.*,1993, Bu *et al.*,1996, Wang *et al.*,2002).

The 1*H*-phenanthro[9,10 - d]imidazole is a promising building block in the field of molecular materials. It has many desirable properties such as good heat stability, ease of introduction into molecules used as chromophores, fluorescent in nature and readily tunable absorption wavelengths.

The study of organic electroluminescent materials and devices (Loy *et al.*, 2002, Adachi *et al.*, 1995) is therefore of great importance. The photophysical, electrochemical and mobility properties of phenanthroimidazole derivatives have been reported (Yuan *et al.*, 2011).

As our research group deals with organic light emitting devices, we are interested in the title compound (I), Figure 1, as a ligand for inorganic complexes.

The dihedral angle between the phenanthrene moiety and the flourobenzene ring is 33.71 (4)° and to that of the benzene ring of methylphenyl is 78.10 (5)°. The dihedral angle between methylphenyl and benzene ring of trifluorobenzene ring is 72.60 (5)°. The maximum deviation of C12 atom from the mean plane of phenanthrotetracyclic system is 0.076 (1)°. The crystal structure is stabilized by C—H···π interactions. One of these, C12–H12···Cg1 is an intramolecular interaction. The other, C26–H26···Cg2 links the molecules into centrosymmetically related pairs across the centre-of-symmetry at (0.5, 0, 0.5), Figure 2. Cg1 and Cg2 are the centres of gravity of the benzene rings C15–C20 and C8–C13 respectively.

### Experimental

A mixture of phenanthrene-9,10-dione (1.0 g, 4.8 mmol), ammonium acetate (1.48 g,19.2 mmol), 4-trifluoromethylbenzaldehyde (0.83 g, 4.8 mmol) and 4-methyl aniline (2.56 g, 24 mmol) were refluxed in ethanol (20 ml) at 80°C. The reaction was monitored by TLC and purified by column chromotography using petroleum ether: ethyl acetate (9:1) as the eluent. Yield: 0.69 g (52%). The compound was dissolved in dimethyl sulfoxide and allowed and slow evaporation produced crystal suitable for X-ray diffraction.

### Refinement

All the hydrogen atoms were geometrically fixed and allowed to ride on their parent atoms with C—H = 0.93 - 0.96 Å and *U*_iso_(H) = 1.3Ueq(C).

The methyl group attached to atom C28 was refined as 6 half hydrogen atoms since a diiference map did not reveal any distinct peaks.

A difference map in the plane of the F atoms of the CF_3_ revealed 3 distinct peaks with evidence of oscillation around the C-C bond connecting the CF_3_ group to the main molecule. The highest difference map peaks were located in the vicinity of the F atoms. Attempts were made to obtain a disordered model but none were satisfactory. These distinct maxima in the difference map were used as the starting positions of F atoms despite problem with thermal parameters during th refinement refinement.

The crystal was a non-merohedral twin. Refinement was carried out using BASF and HKLF 5. The twin data was obtained from PLATON TwinRotMat function, (Spek, 2009).

The twin component ratio is 0.961/0.039.

### Crystal data

**Table:**

C_29_H_19_F_3_N_2_	*Z* = 2
*M**_r_* = 452.46	*F*(000) = 468
Triclinic, *P*1	*D*_x_ = 1.332 Mg m^−^^3^
Hall symbol: -P 1	Mo *K*α radiation, λ = 0.71073 Å
*a* = 8.113 (3) Å	Cell parameters from 6741 reflections
*b* = 11.733 (5) Å	θ = 2.6–27.5°
*c* = 12.713 (2) Å	µ = 0.10 mm^−^^1^
α = 76.397 (1)°	*T* = 293 K
β = 73.490 (2)°	Block, colourless
γ = 86.185 (5)°	0.35 × 0.30 × 0.25 mm
*V* = 1127.7 (7) Å^3^	

### Data collection

**Table:**

Bruker Kappa APEXII CCD diffractometer	22540 independent reflections
Radiation source: Rotating Anode	14735 reflections with *I* > 2σ(*I*)
Graphite monochromator	*R*_int_ = 0.000
Detector resolution: 18.4 pixels mm^-1^	θ_max_ = 27.6°, θ_min_ = 2.6°
ω and φ scan	*h* = −10→10
Absorption correction: multi-scan (*SADABS*; Bruker, 2008)	*k* = −15→14
*T*_min_ = 0.967, *T*_max_ = 0.977	*l* = −16→16
22540 measured reflections	

### Refinement

**Table:**

Refinement on *F*^2^	Secondary atom site location: difference Fourier map
Least-squares matrix: full	Hydrogen site location: inferred from neighbouring sites
*R*[*F*^2^ > 2σ(*F*^2^)] = 0.068	H-atom parameters constrained
*wR*(*F*^2^) = 0.236	*w* = 1/[σ^2^(*F*_o_^2^) + (0.1257*P*)^2^ + 0.462*P*] where *P* = (*F*_o_^2^ + 2*F*_c_^2^)/3
*S* = 1.04	(Δ/σ)_max_ < 0.001
22540 reflections	Δρ_max_ = 0.72 e Å^−^^3^
310 parameters	Δρ_min_ = −0.38 e Å^−^^3^
0 restraints	Extinction correction: *SHELXL97* (Sheldrick, 2008), Fc^*^=kFc[1+0.001xFc^2^λ^3^/sin(2θ)]^-1/4^
Primary atom site location: structure-invariant direct methods	Extinction coefficient: 0.022 (2)

### Special details

**Table:**

Geometry. All esds (except the esd in the dihedral angle between two l.s. planes) are estimated using the full covariance matrix. The cell esds are taken into account individually in the estimation of esds in distances, angles and torsion angles; correlations between esds in cell parameters are only used when they are defined by crystal symmetry. An approximate (isotropic) treatment of cell esds is used for estimating esds involving l.s. planes.
Refinement. Refinement of *F*^2^ against ALL reflections. The weighted *R*-factor *wR* and goodness of fit *S* are based on *F*^2^, conventional *R*-factors *R* are based on *F*, with *F* set to zero for negative *F*^2^. The threshold expression of *F*^2^ > σ(*F*^2^) is used only for calculating *R*-factors(gt) *etc*. and is not relevant to the choice of reflections for refinement. *R*-factors based on *F*^2^ are statistically about twice as large as those based on *F*, and *R*- factors based on ALL data will be even larger.

### Fractional atomic coordinates and isotropic or equivalent isotropic displacement parameters (Å^2^)

**Table:**

	*x*	*y*	*z*	*U*_iso_*/*U*_eq_	Occ. (<1)
N1	0.20865 (14)	0.08289 (9)	0.54048 (8)	0.0456 (3)	
N2	0.30849 (14)	−0.01502 (9)	0.40358 (9)	0.0482 (3)	
F1	0.02200 (19)	−0.51343 (12)	0.86107 (14)	0.1730 (7)	
F2	0.2485 (3)	−0.56334 (10)	0.75953 (12)	0.2022 (10)	
F3	0.2496 (2)	−0.49122 (12)	0.88779 (13)	0.1697 (7)	
C1	0.30932 (17)	0.10375 (11)	0.35746 (11)	0.0464 (3)	
C2	0.36035 (17)	0.15914 (12)	0.24009 (11)	0.0486 (3)	
C3	0.4141 (2)	0.09473 (13)	0.15691 (11)	0.0596 (4)	
H3	0.4196	0.0134	0.1774	0.071*	
C4	0.4590 (2)	0.15053 (15)	0.04533 (12)	0.0721 (5)	
H4	0.4975	0.1074	−0.0098	0.086*	
C5	0.4467 (2)	0.27171 (15)	0.01490 (13)	0.0788 (5)	
H5	0.4732	0.3096	−0.0609	0.095*	
C6	0.3962 (2)	0.33553 (14)	0.09526 (13)	0.0706 (5)	
H6	0.3908	0.4168	0.0731	0.085*	
C7	0.35215 (18)	0.28235 (12)	0.21037 (12)	0.0545 (4)	
C8	0.29676 (19)	0.34923 (12)	0.29833 (13)	0.0565 (4)	
C9	0.2970 (2)	0.47253 (13)	0.27083 (16)	0.0762 (5)	
H9	0.3272	0.5118	0.1954	0.091*	
C10	0.2539 (3)	0.53619 (14)	0.35226 (17)	0.0856 (6)	
H10	0.2552	0.6177	0.3318	0.103*	
C11	0.2086 (2)	0.48010 (15)	0.46402 (17)	0.0793 (5)	
H11	0.1806	0.5240	0.5188	0.095*	
C12	0.2043 (2)	0.36048 (13)	0.49576 (14)	0.0639 (4)	
H12	0.1731	0.3238	0.5719	0.077*	
C13	0.24672 (18)	0.29210 (11)	0.41417 (12)	0.0518 (4)	
C14	0.25034 (17)	0.16657 (11)	0.43936 (11)	0.0461 (3)	
C15	0.14817 (18)	0.10502 (11)	0.65183 (11)	0.0470 (3)	
C16	−0.02091 (18)	0.13450 (12)	0.69267 (12)	0.0570 (4)	
H16	−0.0972	0.1383	0.6495	0.068*	
C17	−0.0763 (2)	0.15835 (13)	0.79825 (13)	0.0644 (4)	
H17	−0.1906	0.1789	0.8255	0.077*	
C18	0.0345 (2)	0.15247 (12)	0.86492 (12)	0.0600 (4)	
C19	0.2023 (2)	0.12097 (14)	0.82111 (12)	0.0675 (4)	
H19	0.2790	0.1155	0.8643	0.081*	
C20	0.26054 (19)	0.09732 (12)	0.71578 (11)	0.0555 (4)	
H20	0.3746	0.0764	0.6884	0.067*	
C21	0.24755 (16)	−0.02465 (11)	0.51342 (10)	0.0445 (3)	
C22	0.22638 (17)	−0.13928 (11)	0.59353 (10)	0.0454 (3)	
C23	0.09865 (19)	−0.16471 (12)	0.69527 (12)	0.0555 (4)	
H23	0.0233	−0.1060	0.7173	0.067*	
C24	0.0823 (2)	−0.27559 (13)	0.76375 (12)	0.0591 (4)	
H24	−0.0045	−0.2918	0.8312	0.071*	
C25	0.1960 (2)	−0.36339 (13)	0.73183 (12)	0.0576 (4)	
C26	0.3215 (2)	−0.33900 (12)	0.63148 (12)	0.0586 (4)	
H26	0.3970	−0.3978	0.6098	0.070*	
C27	0.33689 (18)	−0.22880 (11)	0.56271 (11)	0.0529 (4)	
H27	0.4224	−0.2138	0.4946	0.063*	
C28	−0.0263 (3)	0.18059 (16)	0.97975 (13)	0.0901 (6)	
H28A	−0.1454	0.2025	0.9946	0.135*	0.50
H28B	0.0403	0.2443	0.9817	0.135*	0.50
H28C	−0.0124	0.1129	1.0360	0.135*	0.50
H28D	0.0671	0.1706	1.0135	0.135*	0.50
H28E	−0.1186	0.1288	1.0265	0.135*	0.50
H28F	−0.0659	0.2603	0.9722	0.135*	0.50
C29	0.1800 (3)	−0.48202 (16)	0.80656 (16)	0.0830 (5)	

### Atomic displacement parameters (Å^2^)

**Table:**

	*U*^11^	*U*^22^	*U*^33^	*U*^12^	*U*^13^	*U*^23^
N1	0.0526 (7)	0.0431 (6)	0.0404 (6)	0.0026 (5)	−0.0128 (5)	−0.0092 (5)
N2	0.0554 (7)	0.0463 (7)	0.0405 (6)	0.0003 (5)	−0.0133 (5)	−0.0057 (5)
F1	0.1158 (11)	0.1110 (11)	0.2194 (16)	−0.0340 (9)	−0.0277 (11)	0.0896 (10)
F2	0.367 (3)	0.0503 (7)	0.1142 (11)	0.0110 (11)	0.0252 (13)	0.0111 (7)
F3	0.2296 (18)	0.1301 (11)	0.1480 (12)	−0.0230 (11)	−0.1189 (13)	0.0590 (10)
C1	0.0484 (8)	0.0454 (8)	0.0450 (8)	0.0007 (6)	−0.0172 (6)	−0.0045 (6)
C2	0.0484 (8)	0.0518 (8)	0.0429 (8)	−0.0007 (6)	−0.0156 (6)	−0.0015 (6)
C3	0.0719 (10)	0.0596 (9)	0.0441 (8)	0.0011 (8)	−0.0168 (7)	−0.0050 (7)
C4	0.0875 (12)	0.0794 (12)	0.0426 (9)	−0.0001 (9)	−0.0152 (8)	−0.0044 (8)
C5	0.0981 (13)	0.0792 (12)	0.0445 (9)	−0.0052 (10)	−0.0149 (9)	0.0097 (9)
C6	0.0820 (12)	0.0586 (10)	0.0581 (10)	−0.0039 (9)	−0.0176 (9)	0.0110 (8)
C7	0.0524 (8)	0.0528 (9)	0.0529 (9)	−0.0007 (7)	−0.0184 (7)	0.0034 (7)
C8	0.0567 (9)	0.0459 (8)	0.0637 (10)	0.0038 (7)	−0.0212 (7)	−0.0019 (7)
C9	0.0920 (13)	0.0489 (9)	0.0813 (12)	0.0034 (9)	−0.0274 (10)	0.0008 (9)
C10	0.1043 (15)	0.0442 (9)	0.1030 (15)	0.0082 (9)	−0.0270 (12)	−0.0106 (10)
C11	0.0918 (13)	0.0540 (10)	0.0950 (14)	0.0075 (9)	−0.0260 (11)	−0.0248 (10)
C12	0.0709 (10)	0.0519 (9)	0.0712 (10)	0.0038 (8)	−0.0226 (8)	−0.0158 (8)
C13	0.0527 (8)	0.0455 (8)	0.0589 (9)	0.0025 (6)	−0.0205 (7)	−0.0095 (7)
C14	0.0468 (8)	0.0449 (8)	0.0466 (8)	0.0016 (6)	−0.0168 (6)	−0.0063 (6)
C15	0.0560 (8)	0.0429 (7)	0.0425 (7)	−0.0014 (6)	−0.0140 (6)	−0.0096 (6)
C16	0.0530 (9)	0.0649 (10)	0.0577 (9)	0.0030 (7)	−0.0184 (7)	−0.0197 (8)
C17	0.0598 (10)	0.0636 (10)	0.0640 (10)	0.0012 (8)	−0.0041 (8)	−0.0196 (8)
C18	0.0795 (11)	0.0528 (9)	0.0442 (8)	−0.0029 (8)	−0.0106 (8)	−0.0116 (7)
C19	0.0757 (11)	0.0799 (11)	0.0527 (9)	0.0025 (9)	−0.0278 (8)	−0.0149 (8)
C20	0.0553 (9)	0.0637 (9)	0.0485 (8)	0.0041 (7)	−0.0178 (7)	−0.0115 (7)
C21	0.0468 (8)	0.0444 (8)	0.0416 (8)	−0.0004 (6)	−0.0135 (6)	−0.0064 (6)
C22	0.0507 (8)	0.0443 (8)	0.0421 (7)	−0.0025 (6)	−0.0162 (6)	−0.0066 (6)
C23	0.0559 (9)	0.0547 (9)	0.0510 (8)	0.0014 (7)	−0.0078 (7)	−0.0114 (7)
C24	0.0620 (9)	0.0619 (10)	0.0449 (8)	−0.0093 (8)	−0.0062 (7)	−0.0037 (7)
C25	0.0696 (10)	0.0521 (9)	0.0487 (9)	−0.0115 (8)	−0.0211 (8)	0.0024 (7)
C26	0.0629 (9)	0.0486 (9)	0.0598 (9)	0.0036 (7)	−0.0161 (8)	−0.0057 (7)
C27	0.0543 (9)	0.0500 (8)	0.0476 (8)	−0.0019 (7)	−0.0085 (7)	−0.0043 (7)
C28	0.1210 (16)	0.0905 (13)	0.0543 (10)	−0.0028 (11)	−0.0119 (10)	−0.0219 (9)
C29	0.1007 (15)	0.0612 (12)	0.0729 (12)	−0.0114 (11)	−0.0208 (11)	0.0111 (10)

### Geometric parameters (Å, º)

**Table:**

N1—C21	1.3781 (17)	C13—C14	1.4319 (18)
N1—C14	1.3909 (16)	C15—C20	1.3698 (18)
N1—C15	1.4398 (16)	C15—C16	1.3739 (19)
N2—C21	1.3222 (15)	C16—C17	1.3780 (19)
N2—C1	1.3785 (16)	C16—H16	0.9300
F1—C29	1.303 (2)	C17—C18	1.388 (2)
F2—C29	1.259 (2)	C17—H17	0.9300
F3—C29	1.291 (2)	C18—C19	1.379 (2)
C1—C14	1.3738 (17)	C18—C28	1.510 (2)
C1—C2	1.4312 (17)	C19—C20	1.3751 (19)
C2—C3	1.3963 (19)	C19—H19	0.9300
C2—C7	1.4077 (19)	C20—H20	0.9300
C3—C4	1.3692 (19)	C21—C22	1.4706 (18)
C3—H3	0.9300	C22—C27	1.3889 (18)
C4—C5	1.388 (2)	C22—C23	1.3906 (18)
C4—H4	0.9300	C23—C24	1.3757 (19)
C5—C6	1.361 (2)	C23—H23	0.9300
C5—H5	0.9300	C24—C25	1.391 (2)
C6—C7	1.4006 (19)	C24—H24	0.9300
C6—H6	0.9300	C25—C26	1.369 (2)
C7—C8	1.466 (2)	C25—C29	1.479 (2)
C8—C9	1.406 (2)	C26—C27	1.3710 (18)
C8—C13	1.419 (2)	C26—H26	0.9300
C9—C10	1.369 (2)	C27—H27	0.9300
C9—H9	0.9300	C28—H28A	0.9600
C10—C11	1.372 (2)	C28—H28B	0.9600
C10—H10	0.9300	C28—H28C	0.9600
C11—C12	1.366 (2)	C28—H28D	0.9600
C11—H11	0.9300	C28—H28E	0.9600
C12—C13	1.4107 (19)	C28—H28F	0.9600
C12—H12	0.9300		
			
C21—N1—C14	106.48 (10)	C19—C18—C28	121.62 (16)
C21—N1—C15	126.81 (10)	C17—C18—C28	121.34 (16)
C14—N1—C15	126.58 (10)	C20—C19—C18	122.35 (14)
C21—N2—C1	104.85 (10)	C20—C19—H19	118.8
C14—C1—N2	111.39 (11)	C18—C19—H19	118.8
C14—C1—C2	122.18 (12)	C15—C20—C19	119.07 (14)
N2—C1—C2	126.42 (12)	C15—C20—H20	120.5
C3—C2—C7	120.44 (12)	C19—C20—H20	120.5
C3—C2—C1	122.02 (13)	N2—C21—N1	112.13 (11)
C7—C2—C1	117.53 (13)	N2—C21—C22	121.79 (11)
C4—C3—C2	120.45 (15)	N1—C21—C22	126.08 (11)
C4—C3—H3	119.8	C27—C22—C23	118.28 (13)
C2—C3—H3	119.8	C27—C22—C21	117.48 (12)
C3—C4—C5	119.65 (16)	C23—C22—C21	124.18 (12)
C3—C4—H4	120.2	C24—C23—C22	120.89 (13)
C5—C4—H4	120.2	C24—C23—H23	119.6
C6—C5—C4	120.40 (14)	C22—C23—H23	119.6
C6—C5—H5	119.8	C23—C24—C25	119.79 (13)
C4—C5—H5	119.8	C23—C24—H24	120.1
C5—C6—C7	121.88 (15)	C25—C24—H24	120.1
C5—C6—H6	119.1	C26—C25—C24	119.57 (13)
C7—C6—H6	119.1	C26—C25—C29	120.74 (15)
C6—C7—C2	117.13 (14)	C24—C25—C29	119.69 (15)
C6—C7—C8	122.83 (14)	C25—C26—C27	120.67 (14)
C2—C7—C8	120.03 (12)	C25—C26—H26	119.7
C9—C8—C13	117.68 (15)	C27—C26—H26	119.7
C9—C8—C7	121.00 (14)	C26—C27—C22	120.79 (13)
C13—C8—C7	121.28 (13)	C26—C27—H27	119.6
C10—C9—C8	121.66 (16)	C22—C27—H27	119.6
C10—C9—H9	119.2	C18—C28—H28A	109.5
C8—C9—H9	119.2	C18—C28—H28B	109.5
C9—C10—C11	120.19 (16)	H28A—C28—H28B	109.5
C9—C10—H10	119.9	C18—C28—H28C	109.5
C11—C10—H10	119.9	H28A—C28—H28C	109.5
C12—C11—C10	120.74 (17)	H28B—C28—H28C	109.5
C12—C11—H11	119.6	C18—C28—H28D	109.5
C10—C11—H11	119.6	H28A—C28—H28D	141.1
C11—C12—C13	120.61 (15)	H28B—C28—H28D	56.3
C11—C12—H12	119.7	H28C—C28—H28D	56.3
C13—C12—H12	119.7	C18—C28—H28E	109.5
C12—C13—C8	119.10 (13)	H28A—C28—H28E	56.3
C12—C13—C14	124.56 (13)	H28B—C28—H28E	141.1
C8—C13—C14	116.29 (13)	H28C—C28—H28E	56.3
C1—C14—N1	105.14 (11)	H28D—C28—H28E	109.5
C1—C14—C13	122.53 (12)	C18—C28—H28F	109.5
N1—C14—C13	132.28 (12)	H28A—C28—H28F	56.3
C20—C15—C16	120.59 (13)	H28B—C28—H28F	56.3
C20—C15—N1	119.52 (12)	H28C—C28—H28F	141.1
C16—C15—N1	119.88 (12)	H28D—C28—H28F	109.5
C15—C16—C17	119.35 (14)	H28E—C28—H28F	109.5
C15—C16—H16	120.3	F2—C29—F3	103.88 (19)
C17—C16—H16	120.3	F2—C29—F1	107.1 (2)
C16—C17—C18	121.58 (15)	F3—C29—F1	101.85 (18)
C16—C17—H17	119.2	F2—C29—C25	115.41 (17)
C18—C17—H17	119.2	F3—C29—C25	113.42 (16)
C19—C18—C17	117.04 (13)	F1—C29—C25	113.81 (17)
			
C21—N2—C1—C14	0.56 (15)	C12—C13—C14—N1	3.7 (2)
C21—N2—C1—C2	−178.25 (12)	C8—C13—C14—N1	−178.95 (13)
C14—C1—C2—C3	−177.56 (13)	C21—N1—C15—C20	74.53 (17)
N2—C1—C2—C3	1.1 (2)	C14—N1—C15—C20	−100.77 (15)
C14—C1—C2—C7	1.5 (2)	C21—N1—C15—C16	−105.97 (15)
N2—C1—C2—C7	−179.81 (12)	C14—N1—C15—C16	78.73 (17)
C7—C2—C3—C4	−0.3 (2)	C20—C15—C16—C17	1.1 (2)
C1—C2—C3—C4	178.76 (14)	N1—C15—C16—C17	−178.39 (11)
C2—C3—C4—C5	−1.6 (2)	C15—C16—C17—C18	−0.5 (2)
C3—C4—C5—C6	2.3 (3)	C16—C17—C18—C19	−0.4 (2)
C4—C5—C6—C7	−1.1 (3)	C16—C17—C18—C28	179.00 (13)
C5—C6—C7—C2	−0.7 (2)	C17—C18—C19—C20	0.7 (2)
C5—C6—C7—C8	−179.74 (15)	C28—C18—C19—C20	−178.69 (13)
C3—C2—C7—C6	1.4 (2)	C16—C15—C20—C19	−0.8 (2)
C1—C2—C7—C6	−177.69 (12)	N1—C15—C20—C19	178.68 (12)
C3—C2—C7—C8	−179.54 (13)	C18—C19—C20—C15	−0.1 (2)
C1—C2—C7—C8	1.4 (2)	C1—N2—C21—N1	0.06 (14)
C6—C7—C8—C9	−4.6 (2)	C1—N2—C21—C22	−179.98 (11)
C2—C7—C8—C9	176.33 (13)	C14—N1—C21—N2	−0.64 (15)
C6—C7—C8—C13	177.54 (14)	C15—N1—C21—N2	−176.70 (12)
C2—C7—C8—C13	−1.5 (2)	C14—N1—C21—C22	179.41 (11)
C13—C8—C9—C10	1.2 (3)	C15—N1—C21—C22	3.3 (2)
C7—C8—C9—C10	−176.67 (16)	N2—C21—C22—C27	28.74 (18)
C8—C9—C10—C11	−0.1 (3)	N1—C21—C22—C27	−151.30 (13)
C9—C10—C11—C12	−0.6 (3)	N2—C21—C22—C23	−148.34 (14)
C10—C11—C12—C13	0.2 (3)	N1—C21—C22—C23	31.6 (2)
C11—C12—C13—C8	1.0 (2)	C27—C22—C23—C24	0.1 (2)
C11—C12—C13—C14	178.22 (14)	C21—C22—C23—C24	177.17 (12)
C9—C8—C13—C12	−1.6 (2)	C22—C23—C24—C25	0.8 (2)
C7—C8—C13—C12	176.25 (13)	C23—C24—C25—C26	−1.1 (2)
C9—C8—C13—C14	−179.10 (13)	C23—C24—C25—C29	178.93 (14)
C7—C8—C13—C14	−1.2 (2)	C24—C25—C26—C27	0.5 (2)
N2—C1—C14—N1	−0.94 (14)	C29—C25—C26—C27	−179.52 (14)
C2—C1—C14—N1	177.93 (11)	C25—C26—C27—C22	0.4 (2)
N2—C1—C14—C13	176.67 (11)	C23—C22—C27—C26	−0.7 (2)
C2—C1—C14—C13	−4.5 (2)	C21—C22—C27—C26	−177.97 (12)
C21—N1—C14—C1	0.93 (13)	C26—C25—C29—F2	−20.8 (3)
C15—N1—C14—C1	177.00 (12)	C24—C25—C29—F2	159.2 (2)
C21—N1—C14—C13	−176.36 (13)	C26—C25—C29—F3	98.9 (2)
C15—N1—C14—C13	−0.3 (2)	C24—C25—C29—F3	−81.2 (2)
C12—C13—C14—C1	−173.14 (14)	C26—C25—C29—F1	−145.31 (19)
C8—C13—C14—C1	4.16 (19)	C24—C25—C29—F1	34.7 (3)

### Hydrogen-bond geometry (Å, º)

Cg1 and Cg2 are the centroids of the C15–C20 and C8–C13 rings, respectively.

**Table:**

*D*—H···*A*	*D*—H	H···*A*	*D*···*A*	*D*—H···*A*
C12—H12···*Cg*1	0.93	2.84	3.698 (2)	155
C26—H26···*Cg*2^i^	0.93	2.86	3.487 (2)	125

Symmetry code: (i) −*x*+1, −*y*, −*z*+1.
